# Machine Learning Models for the Early Real-Time Prediction of Deterioration in Intensive Care Units—A Novel Approach to the Early Identification of High-Risk Patients

**DOI:** 10.3390/jcm14020350

**Published:** 2025-01-08

**Authors:** Dominik Thiele, Reitze Rodseth, Richard Friedland, Fabian Berger, Chris Mathew, Caroline Maslo, Vanessa Moll, Christoph Leithner, Christian Storm, Alexander Krannich, Jens Nee

**Affiliations:** 1 Department of Neurology and Experimental Neurology, Charité—Universitätsmedizin Berlin, 10117 Berlin, Germany; 2 TCC Analytics, Telehealth Competence Center (TCC) GmbH, 22083 Hamburg, Germany; 3 Netcare Limited, Johannesburg 2196, South Africa; 4 Department of Anaesthesiology and Critical Care, University of KwaZulu-Natal, Durban 4001, South Africa; 5 Department of Anesthesiology, Division of Critical Care Medicine, University of Minnesota School of Medicine, Minneapolis, MN 55455, USA; 6 Department of Anesthesiology, Division of Critical Care Medicine, Emory University School of Medicine, Atlanta, GA 30322, USA; 7 Department of Nephrology and Medical Intensive Care, Charité—Universitätsmedizin Berlin, 22083 Berlin, Germany; 8 Experimental and Clinical Research Center (ECRC), Charité—Universitätsmedizin Berlin, 22083 Berlin, Germany

**Keywords:** deterioration, real-time prediction, machine learning, ICU, high-risk patients

## Abstract

**Background** Predictive machine learning models have made use of a variety of scoring systems to identify clinical deterioration in ICU patients. However, most of these scores include variables that are dependent on medical staff examining the patient. We present the development of a real-time prediction model using clinical variables that are digital and automatically generated for the early detection of patients at risk of deterioration. **Methods** Routine monitoring data were used in this analysis. ICU patients with at least 24 h of vital sign recordings were included. Deterioration was defined as qSOFA ≥ 2. Model development and validation were performed internally by splitting the cohort into training and test datasets and validating the results on the test dataset. Five different models were trained, tested, and compared against each other. The models were an artificial neural network (ANN), a random forest (RF), a support vector machine (SVM), a linear discriminant analysis (LDA), and a logistic regression (LR). **Results** In total, 7156 ICU patients were screened for inclusion in the study, which resulted in models trained from a total of 28,348 longitudinal measurements. The artificial neural network showed a superior predictive performance for deterioration, with an area under the curve of 0.81 over 0.78 (RF), 0.78 (SVM), 0.77 (LDA), and 0.76 (LR), by using only four vital parameters. The sensitivity was higher than the specificity for the artificial neural network. **Conclusions** The artificial neural network, only using four automatically recorded vital signs, was best able to predict deterioration, 10 h before documentation in clinical records. This real-time prediction model has the potential to flag at-risk patients to the healthcare providers treating them, for closer monitoring and further investigation.

## 1. Introduction

Risk stratification and prediction represent an integral part of clinical medicine and are useful in directing patient investigation and treatment. The more accurately the current state of a patient can be described or quantified, the more accurate the prediction becomes [1]. The utility of the variables used in risk stratification is mainly determined by two factors: (1) their timely proximity to the patient and the outcome being predicted (patient proximity) and (2) their responsiveness to change (i.e., dynamic or static variables); e.g., a prediction model that uses a “history of coronary heart disease” as a risk factor to predict death from acute myocardial infarction is always going to be inferior to a model that includes a current troponin elevation as a risk factor. Similarly, in a patient with cardiac failure, a cardiac echo performed at the time of hospital admission has much greater predictive value than an echo performed one month prior to admission. Closer timely patient proximity will generally improve the predictive ability of a risk factor, as well as the risk scores that include such variables [1].

In intensive care unit (ICU) patients, predictive machine learning (ML) models have made use of a variety of scoring systems to quantify disease severity and identify clinical deterioration. These include acute physiology and chronic health evaluation (APACHE), systemic inflammatory response syndrome (SIRS), sequential organ failure assessment (SOFA), the quick SOFA (qSOFA), National Early Warning Score (NEWS), Modified Early Warning Score (MEWS), and Pediatric Early Warning Score (PEWS) [2,3,4]. There are also extensions to these scores, such as the Queensland Adult Deterioration Detection System (Q-ADDS), which has been reported to have superior discriminatory power in identifying deteriorating patients compared to non-deteriorating patients [5]. However, considering how rapidly an ICU patient’s clinical condition can change, most of these scores rely on relatively static variables. These static variables include, among others, daily laboratory tests, microbiological culture results, and clinical assessments performed once or twice a day. Among these scores, qSOFA can be calculated by the bedside using only the respiratory rate (rr), systolic blood pressure (sbp), and the presence of an altered mental state—Glasgow Coma Scale (GCS) < 15—and so does not rely on laboratory investigations [6].

Continuous telemetric high-frequency vital sign data from ICU patients (heart rate, blood pressure, respiratory rate (RR), and saturation) are both dynamic and temporally proximate to the patient. Incorporating such high-frequency data into risk scores may improve risk score performance. Recent data support this hypothesis, suggesting that high-frequency data sampling strategies may be superior to traditional models in the detection and prediction of deterioration, for example, due to sepsis [7]. There are further data showing that ML algorithms using high-frequency data can achieve good sepsis prediction [8], and several of these models, which demonstrate high prediction accuracy, have already been published and summarized in a recent systematic review [9].

There are a variety of factors that may cause a patient to deteriorate clinically, the most common being respiratory compromise, heart failure, and infection and suspected sepsis [3]. In this study, we developed a real-time ML prediction model using automatically recorded high-frequency vital sign data to identify deteriorating patients, irrespective of the cause. The goal was to develop a model as an early warning system, able to identify deteriorating patients 10 h before onset. A qSOFA score ≥ 2 was defined as significant clinical deterioration. Our aim was to provide a clinical decision support algorithm able to flag patients at risk of deterioration from any cause (respiratory compromise, infection and suspected sepsis, or heart failure) who may benefit from early intervention. In addition, we sought to develop the model using a parsimonious number of variables to reflect the limited information available in the clinical setting [9,10].

## 2. Methods

**Figure 1:**
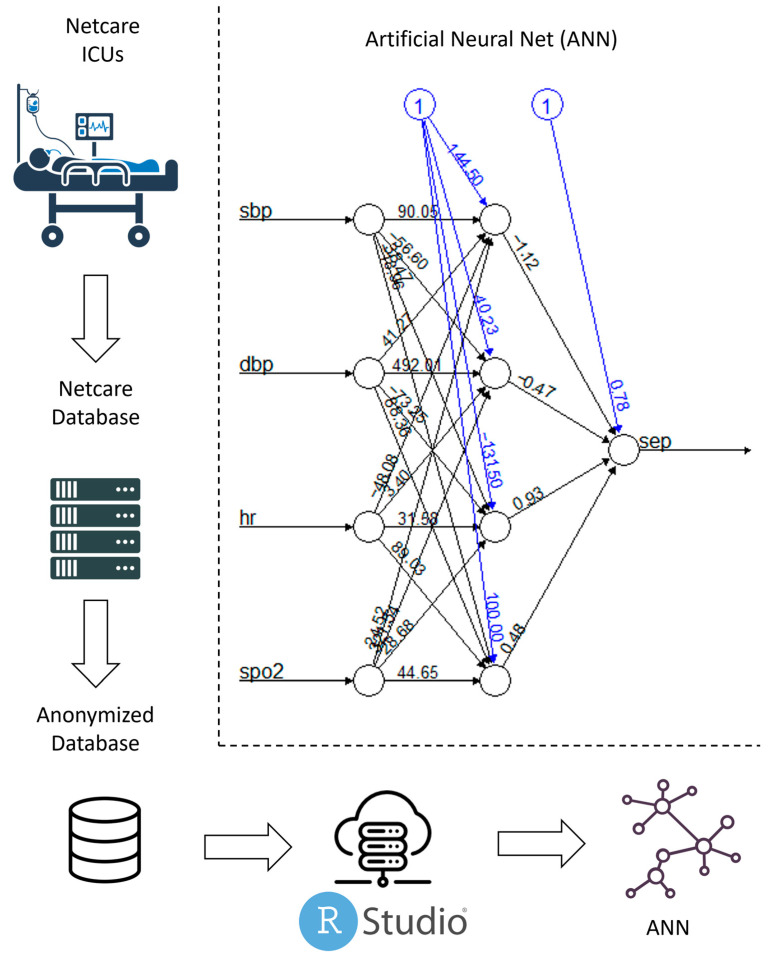
Schematic representation of the data infrastructure and the final best model. Data are transferred from the ICU to a database. The data are then mirrored into an anonymized database. The different models are trained in an RStudio Server environment, from which access to the anonymized database is granted. The final best model is an artificial neural network (ANN). Abbreviations: sbp, systolic blood pressure; dbp, diastolic blood pressure; hr, heart rate; spo2, saturation of peripheral oxygen; ANN, artificial neural network; ICU, intensive care unit.

This article was written in accordance with the TRIPOD statement for the development of prognostic multivariable models [11]. The study used retrospective, fully anonymized electronic data from Netcare Limited “Netcare”, a private healthcare group in South Africa. On 5 February 2023, data were extracted from the newly implemented EMR system, encompassing information from the time period between 2019 and 2022. The process of data cleaning, processing, and analysis was conducted as a joint venture between Netcare South Africa, Charité-Universitätsmedizin Berlin, and Telehealth Competence Center Analytics (TCC Analytics) GmbH Hamburg, Germany. A representation of the data infrastructure is shown in Figure 1. The analysis is part of the RiskML project, approved on 16 August 2020, under EA4/138/22 by the IRB of Charité-Universitätsmedizin Berlin, Berlin, Germany. Informed consent was waived by the committee, and all procedures were followed in accordance with the Declaration of Helsinki of 1975.

### 2.1. Patients

Critical ill adult patients with qSOFA < 2 at the start of observation were included in the analysis if they were admitted to ICU and had at least 24 h of vital sign recordings (diastolic, systolic, and mean blood pressure, respiratory rate, oxygen saturation, heart rate, and temperature) and had a recorded GCS. The GCS, respiratory rate, and systolic blood pressure were used to calculate the deterioration, which served as the outcome for the training of the risk prediction models.

### 2.2. Data Preprocessing

**Figure 2:**
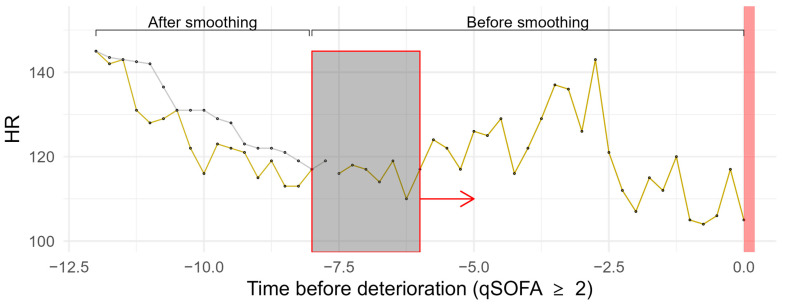
Exemplary presentation of the sliding median window of the heart rate of a patient. The window the size of two hours (red box) is shifted over the course of the measurements. Within the window, the median heart rate is calculated to smoothen the curve. The gray curve shows the smoothened curve. The yellow curve represents the original heart rate. The red bar symbolizes the timepoint when the patient deteriorates.

A median filter was used to smooth all the continuous vital sign measurements [12]. A schema of the algorithm is shown in Figure 2. It made use of a two-hour time window and was shifted over the full course of the 24 h of measurement before reaching deterioration.

**Figure 3:**
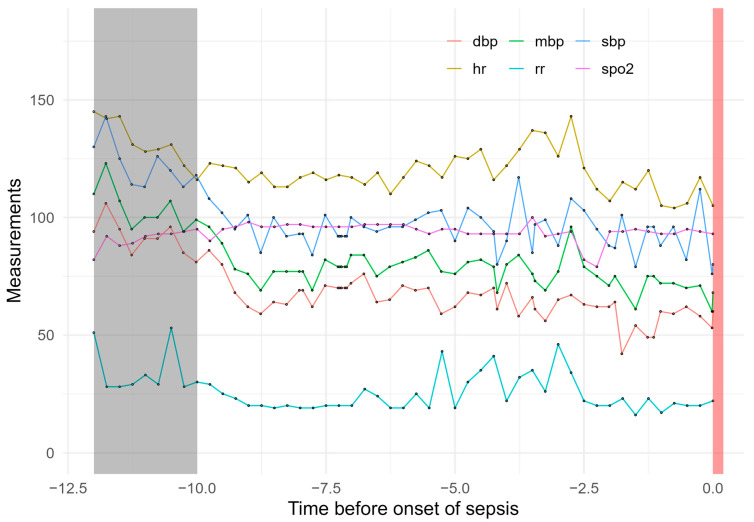
Exemplary presentation of the course of vital signs in one patient with qSOFA ≥ 2. The time period for prediction is marked in gray, and the timepoint when qSOFA ≥ 2 is marked in red. (dbp, diastolic blood pressure; hr, heart rate; mbp, mean arterial blood pressure; rr, respiratory rate; spo2, peripheral capillary oxygen saturation; sbp, systolic blood pressure).

Candidate prediction variables were first chosen based on availability, clinical relevance and use in published prediction models [13,14]. Additionally, we only included automatically recorded high-frequency variables, such as vital signs. Variables matching these criteria were diastolic (dbp), systolic (sbp) and mean blood pressure (mbp), respiratory rate (rr), oxygen saturation (spo2), heart rate (hr), and temperature. An example of these variables is shown in Figure 3. Each variable was mathematically standardized by using a z-transformation. The dataset was randomly split into a training dataset of 100 patients and a test dataset of 73 patients, with a total of 28,348 longitudinal measurements. For a fair comparison of the models, the final selected variables were all the same for all models. In planning the number of cases, we followed Harrell’s guidelines for models, as our sample and the associated events were sufficient for stable models [15].

### 2.3. Machine Learning Methods

An artificial neural network (ANN), a support vector machine (SVM), a random forest (RF), a logistic regression (LR), and a linear discriminant analysis (LDA) were used as machine learning models to predict the binary outcome. All models were trained on the training dataset and tested, evaluated, and compared using the test dataset. A detailed description of the models is provided in Table 1. All analyses were performed using R Statistical Software (v4.2.2; R Core Team 2021) [16].

The area under the curve (AUC) of the receiver operating characteristic (ROC) with its 95% confidence interval (CI) was used as the main evaluation criterion. The intercept and slope of the model’s calibration regression were examined to obtain an impression of model calibration. A slope of 1 with an intercept of 0 reflects perfect calibration. A slope much lower than 1 with an intercept much greater than 0 indicates poor calibration. Models were further compared using sensitivity, specificity, Youden’s J statistic, and the negative and positive predictive value (NPV and PPV). All models were also visually inspected according to their corresponding ROC curve.

## 3. Results

A total of 7156 ICU patients were screened for possible inclusion into the study. Not all patients fulfilled the inclusion criteria and the 24 h observation period for continuous vital signs. Furthermore, clean and complete qSOFA documentation, including respiratory rate, systolic blood pressure, and Glascow coma scale, was required. A reduction in sample size was therefore expected. To the best of our knowledge, the resulting missings are at least missing at random (MAR). Of these, 28,348 longitudinal measurements from 173 patients fulfilled the requirement for full digital and clean documentation. The baseline characteristics of the included patients are provided in Table 2.

This dataset was randomly split into two distinct datasets: a training dataset with 100 patients and a test dataset with 73 patients. All models were trained on the training dataset and evaluated on the test dataset.

Of the candidate prediction variables, only four had a relevant impact on prediction performance. These variables were SBP, RR, SpO2, and HR. The best AUCs for the enumerated variables over the course of time were observed by using the time window 10 h before the deterioration of a patient.

**Figure 4:**
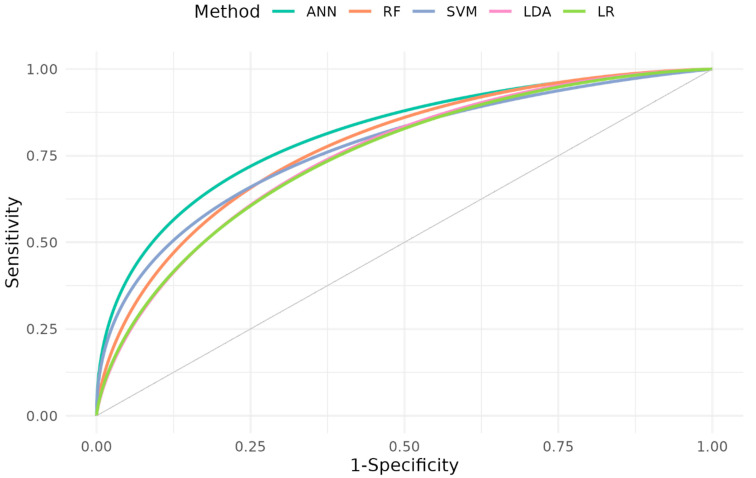
Receiver operating curves (ROCs) of the model comparison 10 h before deterioration.

The results of the evaluation of the model’s prediction performances are summarized in Table 3, and the ROC curves are shown in Figure 4. The best possible prediction performance was achieved by the ANN. The AUC (CI) 0.81 (0.717, 0.912) and the Youden Index 0.52 for the ANN were the best of all the models (AUC range, 0.76–0.78; Youden Index range, 0.48–0.50). The model’s sensitivity (0.85) and the negative predictive value (0.84) were high, whereas the model’s specificity (0.67) and positive predictive value (0.69) were low. The positive likelihood ratio (LR) for the model was 2.56, and the negative LR was 0.22. The calibration regression results (intercept −0.12/slope 1.26) were close to the values of a perfectly calibrated model.

## 4. Discussion

In this analysis, we present different prediction models that use automatically recorded high-frequency data from ICU patients to predict the risk of a patient physiologically deteriorating within the next 10 h. All ML models performed well, but the best model was the ANN with one hidden layer (AUC = 0.81) predicting risk of deterioration 10 h before onset. All other models had a lower AUC compared to the ANN but were comparable to each other. These models also had greater specificity than sensitivity, while the ANN model had greater sensitivity than specificity. The other models also had greater PPVs than the ANN model. For all models, the calibration fell within an acceptable range.

Overall, these results suggest that the ANN model was superior in detecting patients at high risk for deterioration. The low specificity and PPV show that the model might tend towards false positives, whereas the high NPV indicates a low chance of false negatives. With these characteristics the model is a powerful screening tool to detect patients at high risk in a very early stage. The other models are balanced with less false-positive decisions compared to the ANN but therefore have an increased rate of false negatives. As machine learning models can be seen as a clinical decision support tool, lower rates of false positives suggest possible greater utility for the ANN model.

All models made use of only four vital sign parameters, which are automatically measured at high frequency during an ICU stay. This allows for the constant real-time prediction of a patient’s individual risk of deterioration and offers a diagnostic and therapeutic window of approximately 10 h. Most ML prediction models use a high number of static variables. These include laboratory data that are usually processed once a day and manually calculated scores that may be subjective and influenced by human error [21,22]. These factors limit their practical clinical useability, particularly considering the rapid development of the leading causes for deterioration on ICUs, such as respiratory instability, infection and suspected sepsis, and heart failure [23,24,25,26,27,28,29,30]. Any delay in diagnosis will significantly impact mortality [31,32]. Furthermore, by flagging patients at high risk for deterioration by using only a few automatically generated data, the model offers a wide range of applications even outside of the ICU, as many hospitals have digital EMR, including vitals [33,34].

Translating these findings to the bedside, e.g., for the very early risk stratification of sepsis, is of special interest due to high mortality. While many models have been published, few have been able to predict sepsis risk several hours before its onset. A model with an AUC > 0.8 predicting sepsis 4, 6, 8, and 10 h before onset and a second model with an AUC of 0.88 predicting the risk of sepsis for the “next day” have already been developed [21,22]. Other groups have also achieved good sepsis prediction but only up to 4 h before sepsis onset [8,13,14,21,35,36,37,38,39,40]. As previously described, most of these models make use of static data points [36]. It is, therefore, advantageous to have prediction models that can be used as clinical decision support mechanisms that automatically provide alerts within the electronic health record without requiring any additional manual workload [41]. The SSC has strongly recommended the inclusion of sepsis screening in acutely ill patients [41]. However, using a single qSOFA score is not recommended, as the presence of a high qSOFA score may already be indicative of ongoing sepsis. This again highlights the advantages of using high-frequency data as the basis for risk prediction, well in advance of a change in qSOFA score [41]. Further, using a parsimonious number of variables in the ML model, together with fully automated parameter generation, only simplifies the analysis and better reflects clinical routine. However, the model needs validation for risk prediction for deterioration in an external dataset and also for the incidence of sepsis cases predicted at a very early stage.

While the potential benefits in the early identification of sepsis are clear, the model also holds great promise in identifying physiological deterioration due to causes other than sepsis. The algorithm would also quantify progressive physiological deterioration from respiratory or heart failure, acute arrhythmias, or hypovolemia. Therefore, it is important to treat this algorithm as a clinical decision support for flagging those at high risk of deterioration rather than for pointing out the most likely diagnosis.

The limitations of this analysis need to be addressed. First, it is not an observational study with study-related specific data collection. Data are gathered from routine databases, which could lead to several bias types. Second, the aim of our models was to predict, not to investigate, the therapeutic results of the prediction. The actual health outcome could be independent from the initial prediction. Therefore, the model performance and the actual health outcomes should be investigated in the future. Third, due to the limited observation period of 24 h per patient, a lot of information, such as the length of stay, was not included in the analysis. Furthermore, some of the routine documentation is not suitable for the proper categorization of diagnoses and therapies. This corresponds to the target situation for such algorithms but can lead to a relevant bias in the results.

Together with the fact that the data were collected from routine documentation, with limited information on therapy and diagnosis, the potential bias should be further investigated in an external sample.

## 5. Conclusions

The algorithm presented shows the possibilities of making predictions several hours in advance with limited information such as monitoring data. The accuracy achieved is satisfactory, although the risk of bias when using routine data is very high. Further studies must show how robust these approaches are in routine clinical operations, although algorithms offer promising possibilities as new tools.

## Figures and Tables

**Table 1:** Models and respective parameter settings that were used to train and test the models to predict qSOFA ≥ 2.

Method	Parameters		R Package	Version
Logistic regression			stats [16]	3.6.3
Artificial neural network	Algorithm:	Resilient backpropagation +(PROP+)	neuralnet [17]	1.44.2
	No. of hidden layers:	1		
	Stopping criterion:	Threshold 0.01		
	Maximal no. of training steps:	100,000		
	Error function:	Sum-of-squares error		
	Activation function:	Logistic function		
	Output function:	Simple threshold		
Support vector machine	Kernel:	Radial basis kernel	e1071 [18]	1.7–12
	γ:	1/4		
	Cost of constraints:	1		
	Maximum margin error:	0.5		
	Tolerance of termination criterion:	0.001		
	Ɛ in the loss function:	0.1		
Random forest	No. of trees:	500	ranger [19]	0.14.1
	No. of variables for splitting:	2		
	Splitting criterion:	Gini index		
	Minimal node size:	1		
	Depth of each tree:	Unlimited		
	Selection of observations:	Sampling with replacement		
Linear discriminant analysis	Initial means of groups:	Estimated from data	MASS [20]	7.3–58.1
	Initial variances of groups:	Estimated from data		

**Table 2:** Baseline characteristics of the cohort. Patients at risk of deterioration are defined as qSOFA ≥ 2.

	Cohort Development Phase		Model Development Phase	
	overall		training dataset	test dataset
	qSOFA ≥ 2	qSOFA < 2		
n	76	97	100	73
age in years (sd)	63.6 (20.3)	62.3 (16.5)	63.7 (18.9)	61.8 (17.3)
gender, female/male (%)	32/44 (42.1/57.9)	44/53 (45.3/54.7)	47/53 (47.0/53.0)	29/44 (39.7/60.3)
circulatory or respiratory diagnosis, yes/no (%)	40/36 (52.6/47.4)	46/51 (47.4/52.6)	47/53 (47.0/53.0)	39/34 (53.4/46.6)

Metric variables described by mean (sd) and categorical variables described by absolute frequency (relative frequency).

**Table 3:** Prediction performances of trained models on the test dataset.

Method	ANN	RF	SVM	LDA	LR
AUC (CI)	0.814 (0.717, 0.912) *p* = 0.002	0.781 (0.674, 0.887) *p* = 0.005	0.778 (0.670, 0.886) *p* = 0.006	0.765 (0.652, 0.877) *p* = 0.011	0.762 (0.650, 0.875) *p* = 0.011
Sensitivity	0.853	0.706	0.706	0.735	0.735
Specificity	0.667	0.795	0.769	0.744	0.744
PPV	0.690	0.750	0.727	0.714	0.714
NPV	0.839	0.756	0.750	0.763	0.763
Youden’s J statistic	0.52	0.501	0.475	0.479	0.479
Calibration intercept	−0.123	0.126	−0.033	0.117	0.095
Calibration slope	1.259	0.796	1.171	0.824	0.881
Cut-off	0.335	0.435	0.443	0.395	0.409
LR+	2.559	3.441	3.059	2.868	2.868
LR-	0.221	0.37	0.382	0.356	0.356

ANN, artificial neural network; RF, random forest; SVM, support vector machine; LDA, linear discriminant analysis; LR, logistic regression; AUC, area under curve; CI, 95% confidence interval; PPV, positive predictive value; NPV, negative predictive value; LR, likelihood ratio.

## Data Availability

The data presented in this study are available on request from the corresponding author due to patient privacy concerns and compliance with European data protection policies. These restrictions are in place to safeguard the confidentiality and personal information of study participants, in accordance with the General Data Protection Regulation (GDPR) and other applicable European data protection laws.
